# [18F]Fluorodeoxyglucose accumulation as a biological marker of hypoxic status but not glucose transport ability in gastric cancer

**DOI:** 10.1186/1756-9966-32-34

**Published:** 2013-05-29

**Authors:** Ryusuke Takebayashi, Kunihiko Izuishi, Yuka Yamamoto, Reiko Kameyama, Hirohito Mori, Tsutomu Masaki, Yasuyuki Suzuki

**Affiliations:** 1 Department of Gastroenterological Surgery, Kagawa University 1750-1, Ikenobe, Miki, Kita, Kagawa 761-0793, Japan; 2 Department of Radiology, Kagawa University 1750-1, Ikenobe, Miki, Kita, Kagawa 761-0793, Japan; 3 Department of Internal Medicine of Gastroenterology and Neurology, Faculty of Medicine, Kagawa University 1750-1, Ikenobe, Miki, Kita, Kagawa 761-0793, Japan; 4 Department of Gastroenterological Surgery, Federation of Public Services and Affiliated Personnel Aid Associations, Takamatsu Hospital, 4-18 Tenjinmae, Takamatsu, Kagawa 760-0018, Japan

**Keywords:** 18-Fluorodeoxyglucose, Positron emission tomography, Gastric cancer, Glucose transporter-1, Hypoxia-inducible factor 1α

## Abstract

**Background:**

The use of [18F] 2-fluoro-2-deoxy-D-glucose positron emission tomography (FDG-PET) for detection of gastric cancer is often debated because FDG uptake varies for each patient. The purpose of this study was to clarify the molecular mechanisms involved in FDG uptake.

**Material and methods:**

Fifty patients with gastric cancer who underwent FDG-PET and gastrectomy were studied. Snap-frozen tumor specimens were collected and examined by real-time PCR for relationships between maximum standardized uptake value (SUV) and mRNA expression of the following genes: glucose transporter 1 (GLUT1), hexokinase 2 (HK2), hypoxia-inducible factor 1α (HIF1α), and proliferating cell nuclear antigen (PCNA).

**Results:**

Tumor size was the only clinicopathological parameter that significantly correlated with SUV. Transcripts for the genes evaluated were about three-fold higher in malignant specimens than in normal mucosa, although only HIF1α was significantly correlated with SUV. When divided into intestinal and non-intestinal tumors, there was a significant correlation between SUV and tumor size in intestinal tumors. Interestingly, the weak association between SUV and HIF1α expression in intestinal tumors was substantially stronger in non-intestinal tumors. No correlation was found between SUV and mRNA expression of other genes in intestinal or non-intestinal tumors.

**Conclusion:**

SUV was correlated with HIF1α, but not PCNA, HK2, or GLUT1 expression. FDG accumulation could therefore represent tissue hypoxia rather than glucose transport activity for aggressive cancer growth.

## Background

Radiology examinations provide important information for cancer treatment, and [18F] 2-fluoro-2-deoxy-D-glucose positron emission tomography (FDG-PET) differs from conventional imaging through its use of cellular metabolic characteristics to detect a variety of tumors and metastases [1,2]. FDG-PET detection rates tended to vary widely for gastric cancer, however, with 0–44% detection in early stages and 34–94% detection in advanced stages [1,3-5]. Pseudolesions from physiological FDG uptake prevent a more precise diagnosis [6]. Moreover, signet ring cell carcinoma was reported to significantly lower the standardized uptake value (SUV) of FDG compared to papillary or tubular adenocarcinomas [1,7,8]. The usefulness of FDG-PET detection for gastric cancer is thus a matter of debate.

Besides detecting tumors based on absolute value, FDG-PET can also assess the response to chemotherapy based on relative values before and after cancer treatment [1]. Previous studies have suggested a significant association between the metabolic changes observed by FDG-PET and clinical or histopathological response [9-11]. One report in particular predicted patient prognoses by detecting early changes in glucose uptake after chemotherapy, which could help prevent the continuation of ineffective treatments. Ott et al. found that a reduction in FDG uptake of more than 35% for metabolic responders predicted a favorable response in gastric cancer patients two weeks after initiation of chemotherapy [11], while metabolic non-responders or FDG non-avid tumors received an unfavorable prognosis.

Cancer cells theoretically require a greater amount of glucose consumption than healthy tissue because of increased cell division [12,13] or anaerobic respiration in tumors [14]. Many cancers increase glucose transport through glucose transporter 1 (GLUT1) and glucose phosphorylation by hexokinase (HK) [15-17]. A correlation between FDG uptake and GLUT1 expression has been found in gastric cancer patients [1,3,7,8], but these studies were conducted by non-quantitative immunohistochemistry analysis, such as negative or positive staining that can vary by evaluator. We therefore evaluated the expression of glucose metabolism-related proteins through quantitative reverse-transcription polymerase chain reaction (qRT-PCR) and compared the results to maximum SUV of FDG-PET. In addition, we also analyzed the expression of proliferating cell nuclear antigen (PCNA) as a valid marker of proliferation [18] and hypoxia-inducible factor 1 alpha (HIF1α) as a marker of hypoxia [19] to elucidate either of these mechanisms, i.e., tumor proliferation or tumor hypoxia, contribute to FDG uptake. We then discuss the significance and difficulties involved with the clinical application of FDG-PET in gastric cancer due to FDG uptake mechanisms.

## Materials and methods

### Patients

This retrospective study involved 50 patients (29 male and 21 female; mean age ± standard error of measurement [SEM], 65.8 ± 1.4 years) with gastric cancer who underwent same FDG-PET system before gastrectomy in Kagawa University from July 2005 to March 2010. Tumor specimens were snap-frozen at the time of surgery, and stored at −80°C. Participants were divided into 25 cases of intestinal tumors and 25 cases of non-intestinal tumors based on histopathological diagnoses. When focal FDG uptake was not found in the stomach, SUV was calculated from a lesion determined by histology results after gastrectomy. The International Union Against Cancer staging system was used to determine clinicopathological parameters associated with FDG uptake. The protocol was approved by the institutional review board of our institution, and all patients provided written informed consent.

### FDG-PET imaging

FDG-PET images were acquired with a PET scanner (ECAT EXACT HR+, Siemens/CTI, Knoxville, TN, USA). Patients fasted at least five hours before FDG injection. Images were reviewed on a Sun Microsystems workstation (Siemens/CTI) along transverse, coronal, and sagittal planes with maximum intensity projection images. The images were then interpreted independently by two experienced nuclear medicine physicians blinded to the clinical data. Tumor lesions were identified as areas of focally increased FDG uptake exceeding that of surrounding normal tissue. A region of interest was placed over each lesion to include the highest levels of radioactivity. Maximum SUV was calculated with the following formula: SUV = cdc/(di/w), wherein cdc is the decay-corrected tracer tissue concentration (Bq/g), di is the injected dose (Bq), and w is the patient’s body weight (g).

### Immunohistochemical staining

Immunohistochemical staining was performed to determine GLUT1 and HK2 levels in gastric cancer tumors. Briefly, resected specimens were fixed in 10% buffered formalin solution, embedded in paraffin, and sectioned at a thickness of 4 μm. Slides were then incubated overnight at room temperature with primary rabbit polyclonal antibody against GLUT1 (1:200) or HK2 (1:100). Avidin-biotin-peroxidase complex staining was performed according to the manufacturer’s instructions (Santa Cruz Biotechnology, CA, USA). Finally, nuclei were counterstained with hematoxylin [20].

### Real-time PCR

Total RNA was isolated from specimens by guanidinium isothiocyanate-acid phenol extraction and quantified by absorbance at 260 nm. Total RNA (1 μg) was used for reverse transcription, and the resulting cDNA was analyzed by real-time PCR with Power SYBR Green PCR Master Mix and ABI Prism 7000 (Applied Biosystems, Foster, CA, USA). Target-specific oligonucleotide primers and probes were previously described [20,21]. 18S rRNA was used as an endogenous control. Primers and probes for 18S rRNA were obtained in a Pre-Developed TaqMan Assay Reagent kit (Applied Biosystems, Stockholm, Sweden).

### Statistical analysis

Data are expressed as mean ± SEM. Paired SUV results were compared by student’s *t*-test. Multiple one-way analysis of variance was used to assess differences in mRNA levels. Correlation analyses were performed with Spearman’s correlation analysis test. P<0.05 was considered statistically significant.

## Results

### Relationship between mean SUV and clinicopathological data in gastric cancer

**Figure 1:**
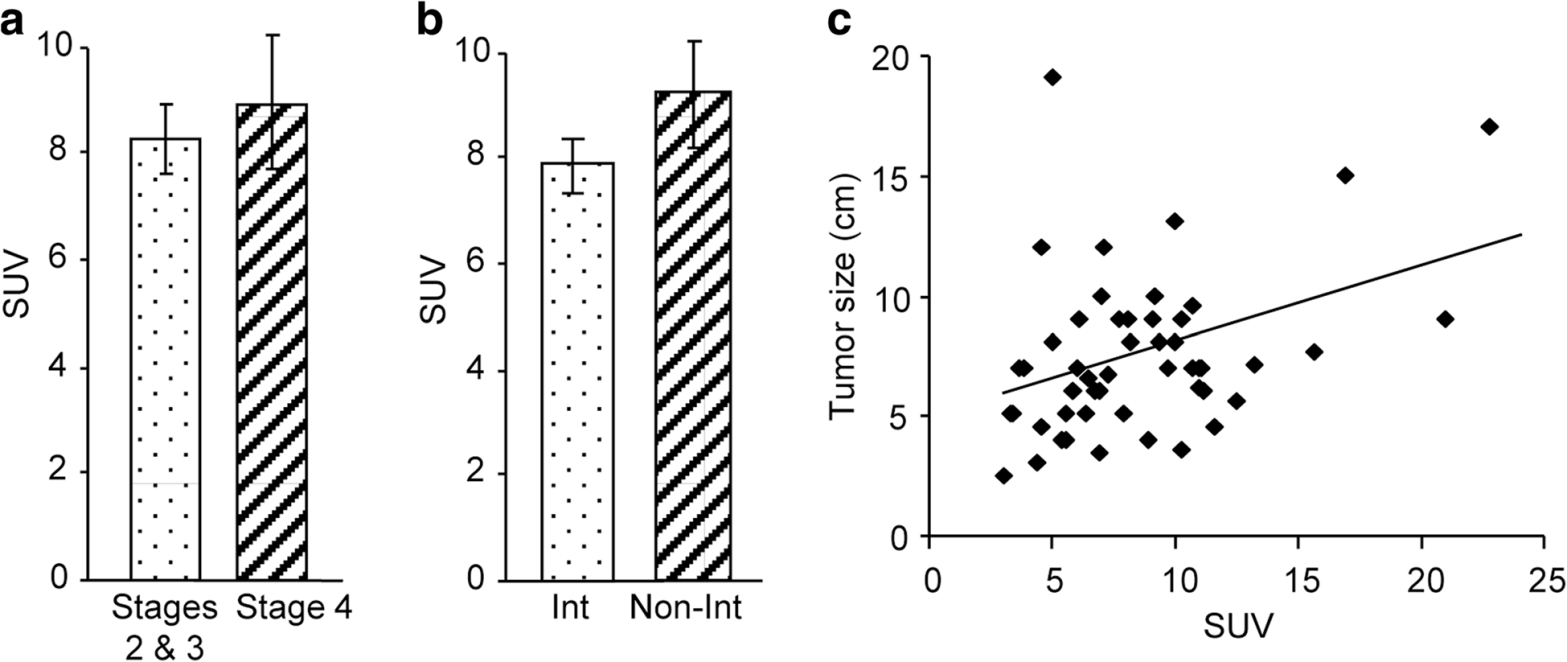
**Relationship between mean standardized uptake value and clinicopathological data in gastric cancer.** (**a**) Mean standardized uptake value (SUV) in stage 4 gastric cancer patients was not significantly higher than in stage 2 and stage 3 patients. (**b**) Mean SUV in intestinal tumors was not significantly greater than in non-intestinal tumors. (**c**) Spearman’s correlation analysis revealed a significant correlation between tumor size and mean SUV (rs = 0.33, P < 0.05). Values are expressed as mean ± SEM. Int; Intestinal Type, Non-Int; Non-intestinal Type, SUV; Standardized Uptake Value.

Of the 50 gastric cancer lesions, 45 showed focally increased FDG uptake. The majority of patients had advanced gastric cancer and a mean tumor size of 7.5 ± 0.5 cm, with 16 cases classified as stage 4. The mean SUV of stage 4 patients was 9.0 ± 1.3, while mean SUV of stage 2 and stage 3 patients combined was 8.3 ± 0.6 (Figure 1a). When tumors were divided into intestinal and non-intestinal tumors, mean SUVs were 7.8 ± 0.7 and 9.2 ± 1.0, respectively (Figure 1b). When divided by median lymph node metastasis, 22 cases had less than three and 28 cases had three or more; mean SUVs were not significant at 9.4 ± 1.0 and 7.8 ± 0.7, respectively. When divided by maximum median tumor diameter, 22 cases were less than 7.0 cm and 28 cases were 7.0 cm or greater; mean SUVs were 7.0 ± 0.6 and 9.7 ± 0.9, respectively (P < 0.05).

These results indicate that SUV was not dependent on the number of lymph node metastases or cancer stage. Maximum tumor diameter was the only parameter with a significant difference. To more precisely determine its correlation with SUV, we carried out quantitative analysis (Figure 1c). Spearman’s correlation analysis indicated a possible relationship between the factors (rs = 0.33, P < 0.05).

### Expression of glucose transporter and glucose metabolizing enzymes in gastric cancer

**Figure 2:**
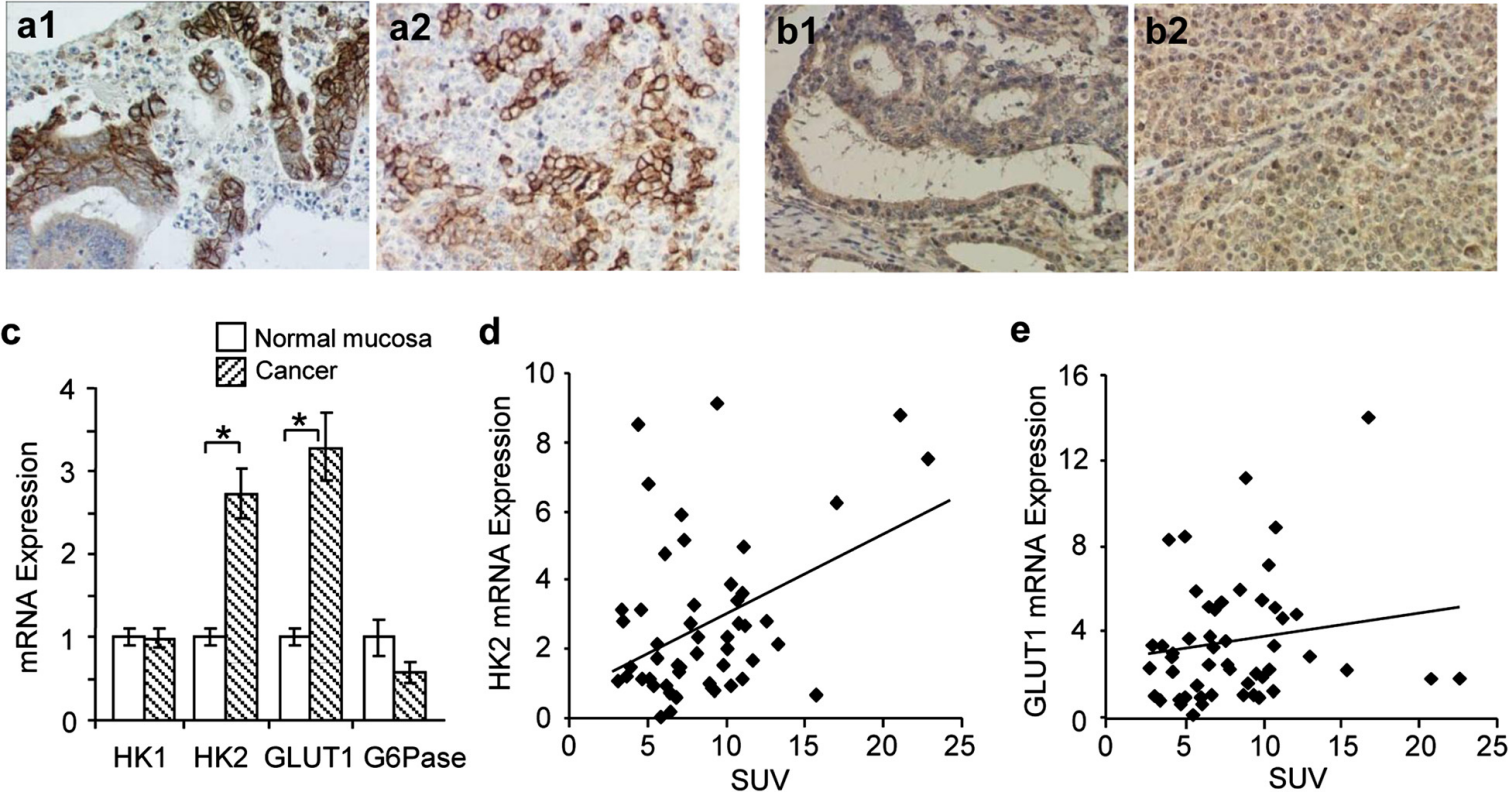
**Expression of glucose transporter and glucose metabolizing enzymes in gastric cancer.** (**a**) Glucose transporter 1 (GLUT1) staining was strong in the cell walls of tubular (a1) and poorly differentiated adenocarcinomas (a2). (**b**) Staining for hexokinase 2 (HK2) was seen in the cytoplasm of tubular (b1) and poorly differentiated adenocarcinomas (b2). (**c**) Increased mRNA expression of glucose metabolism-related proteins was observed with HK2 and GLUT1, but not HK1 and Glucose-6-phosphatase (G6Pase). (**d**-**e**) Spearman’s correlation analysis found no association between standardized uptake value (SUV) and HK2 (**d**) or GLUT1 (**e**) mRNA expression. Values are expressed as mean ± SEM. *P < 0.05. GLUT1; Glucose transporter 1, G6Pase; Glucose-6-phosphatase, HK1; Hexokinase 1, HK2; Hexokinase 2, SUV; Standardized Uptake Value.

GLUT1 staining was seen in the cell walls, while HK2 staining was observed in the cytoplasm, of tubular (Figure 2a1, 2b1) and poorly differentiated (Figure 2a2, 2b2) adenocarcinomas. Based on these results, specimens were evaluated by qRT-PCR to determine the expression of glucose metabolism-related genes (HK1, HK2, GLUT1, and glucose-6-phosphatase (G6Pase)). HK2 and GLUT1 levels were three-fold higher in cancerous tissue than in normal mucosa (P < 0.001) (Figure 2c). G6Pase is a gluconeogenic enzyme in the liver that reverses the reaction metabolized by HK (glucose to glucose-6-phosphate) [22]. Its expression appeared to decrease in cancerous tissue, but not to a significant degree. In spite of the high levels, no significant correlation was observed between SUV and HK2 (Figure 2d) or GLUT1 (Figure 2e) expression. The glucose metabolic pathway in cancerous tissues may be too complicated to regulate with the alteration of a single molecule.

### Relationship between mean SUV and HIF1α or PCNA expression in gastric cancer

**Figure 3:**
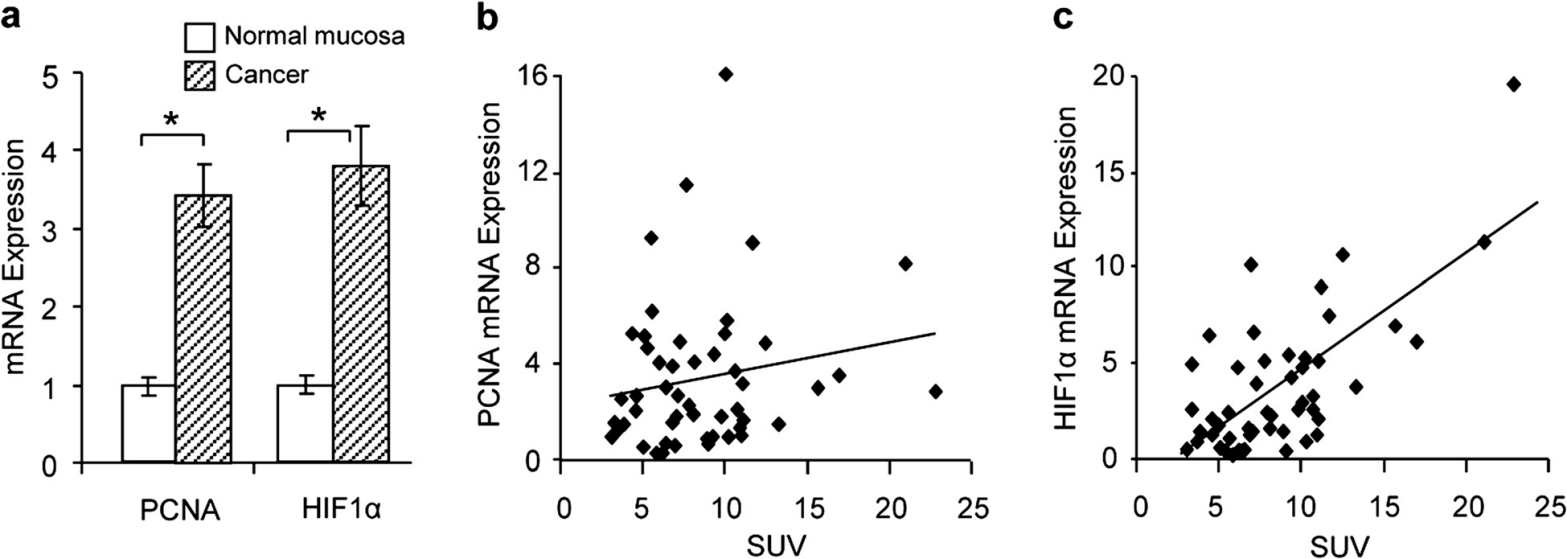
**Relationship between mean standardized uptake value and hypoxia**-**inducible factor 1α or proliferating cell nuclear antigen expression in gastric cancer.** (**a**) mRNA levels for both genes were about three-fold higher in malignant specimens than in normal mucosa (P < 0.001). (**b**) Spearman’s correlation analysis found no association between standardized uptake value (SUV) and proliferating cell nuclear antigen (PCNA) mRNA expression. (**c**) A significant correlation was found between SUV and hypoxia-inducible factor 1α (HIF1α) mRNA expression (r = 0.53, P < 0.01). Data are expressed as mean ± SEM *P < 0.05. HIF1α; Hypoxia-inducible factor 1α, PCNA; Proliferating cell nuclear antigen, SUV; Standardized Uptake Value.

To determine whether tumor proliferation or tumor hypoxia contributes to FDG uptake, PCNA expression was analyzed as a proliferation marker and HIF1α expression as a hypoxia marker. The mRNA levels for both genes were about three-fold higher in cancerous cells than in normal mucosa (P < 0.001) (Figure 3a). To more precisely determine the association of SUV with PCNA and HIF1α mRNA expression, their correlation was quantitatively analyzed. There was no correlation between PCNA expression and SUV (Figure 3b), but HIF1α expression was correlated to SUV by Spearman’s correlation analysis (rs = 0.53, P < 0.01) (Figure 3c). There was no correlation between PCNA expression and HIF1α expression (data not shown).

### Expression of HK1, HK2, GLUT1, and G6Pase mRNA levels in intestinal and non-intestinal gastric cancers

**Figure 4:**
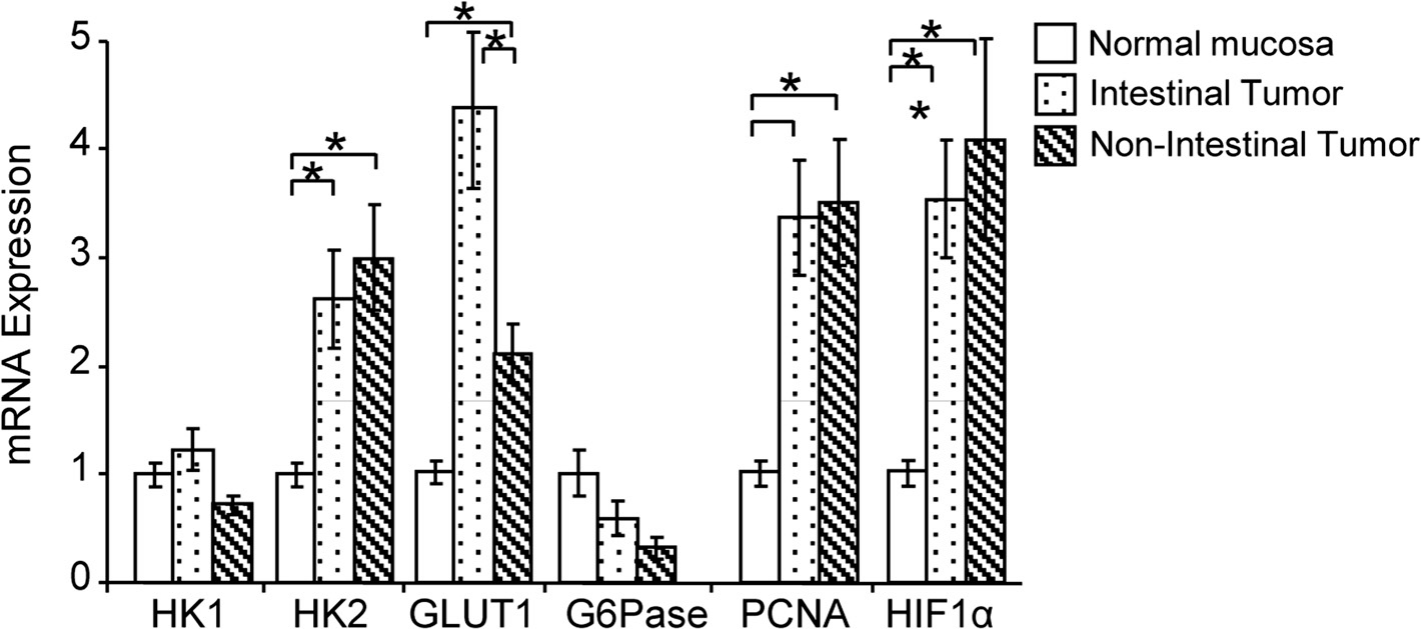
**Expression of glucose metabolism**-**related proteins in intestinal and non**-**intestinal gastric cancers.** Hexokinase 1 (HK1) mRNA levels were similar to those in normal mucosa, while HK2 mRNA levels were higher in both intestinal and non-intestinal gastric cancers (P < 0.01). Glucose transporter 1 (GLUT1) expression increased more in intestinal tumors than in normal mucosa (P < 0.01), but were unchanged in non-intestinal tumors. Glucose-6-phosphatase (G6Pase) expression decreased, but the difference was not significant. The mRNA expression of proliferating cell nuclear antigen (PCNA) and hypoxia-inducible factor 1α (HIF1α) increased more than three-fold compared to normal mucosa (P < 0.01). Data are expressed as mean ± SEM *P < 0.05 (ANOVA). GLUT1; Glucose transporter 1, G6Pase; Glucose-6-phosphatase, HIF1α; Hypoxia-inducible factor 1α, HK1; Hexokinase 1, HK2; Hexokinase 2, PCNA; Proliferating cell nuclear antigen, SUV; Standardized Uptake Value.

Although HK1 mRNA levels were similar, HK2 mRNA levels were higher in both specimen types compared to normal mucosa (P < 0.01). GLUT1 expression was significantly higher in intestinal specimens than in normal mucosa (P < 0.01), but was unchanged in non-intestinal specimens (Figure 4). PCNA and HIF1α expression increased three-fold in intestinal tumors (P < 0.01) compared to normal mucosa.

### Correlation between mean SUV and tumor size, HIF1α mRNA levels, or PCNA mRNA levels in intestinal and non-intestinal gastric cancers

**Figure 5:**
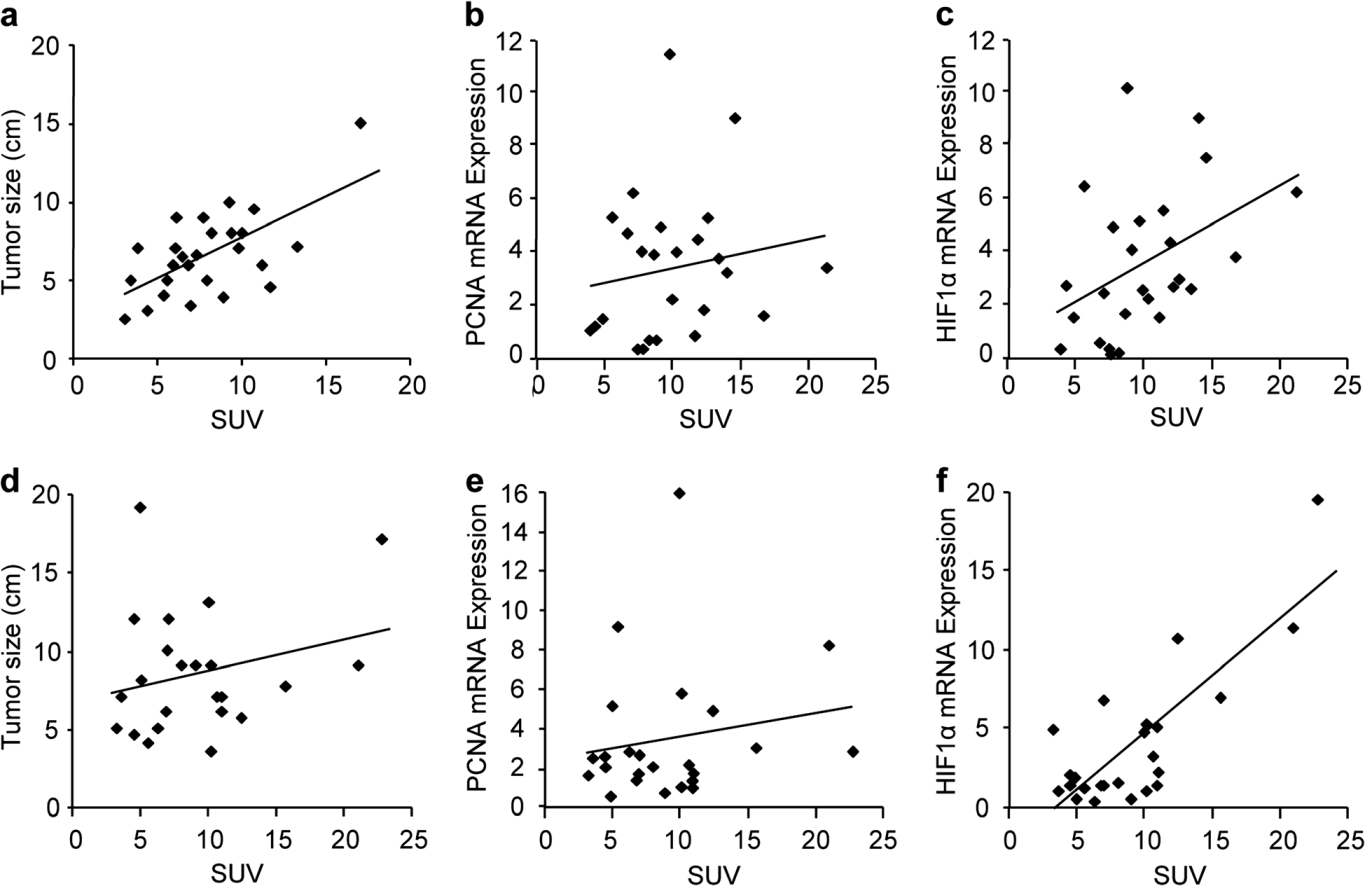
**Correlation between mean standardized uptake value and tumor size**, **hypoxia**-**inducible factor 1α mRNA levels**, **or proliferating cell nuclear antigen mRNA levels in intestinal and non**-**intestinal gastric cancers.** (**a**) Spearman’s correlation analysis indicated a possible correlation between standardized uptake value (SUV) and tumor size in intestinal cancers (rs= 0.50, P < 0.05). (**b**) No association was found between SUV and proliferating cell nuclear antigen (PCNA) mRNA expression. (**c**) A weak association was observed between SUV and hypoxia-inducible factor 1α (HIF1α) mRNA expression (rs = 0.48, P < 0.05). (**d**) In non- intestinal cancer specimens, SUV was not correlated to tumor size. (**e**) No association was found between SUV and PCNA expression. (**f**) A significant correlation between SUV and HIF1α mRNA expression was observed (rs = 0.56, P < 0.01). Data are expressed as mean ± SEM. *P < 0.05. HIF1α; Hypoxia-inducible factor 1α, PCNA; Proliferating cell nuclear antigen, SUV; Standardized Uptake Value.

To examine factors associated with SUV in intestinal and non-intestinal gastric cancers, their correlation was quantitatively analyzed. Spearman’s correlation analysis indicated a possible relationship between SUV and tumor size in intestinal specimens (rs = 0.50, P < 0.05) (Figure 5a), but not non-intestinal specimens (Figure 5d). The correlation between HK2 or GLUT1 expression and SUV did not find in both cancers (data not shown). There was no correlation between SUV and PCNA mRNA expression in either cancer type (Figure 5b and 5e). Interestingly, the weak association between SUV and HIF1α mRNA expression in intestinal specimens (rs = 0.48, P < 0.05) (Figure 5c) was stronger in non-intestinal specimens (rs = 0.56, P < 0.01) (Figure 5f).

## Discussion

FDG-PET has been used to not only detect cancerous lesions, but also predict therapeutic response after chemotherapy [1,11,23]. There are several possible mechanisms behind its ability to reveal malignant potential or cancer cell activity. Our results found that SUV in stage 4 gastric cancer patients was no higher than in stage 2 and stage 3 patients, and the main tumor SUV did not reflect the number of lymph node metastases. Only tumor size was associated with SUV, a correlation also reported in breast, pancreatic, and colorectal cancers [20,24,25]. These finding narrow the FDG-PET mechanism possibilities by suggesting that SUV reflects tumor size rather than tumor cell activity for each cancer stage.

### Over expression of glucose metabolism-related protein in tumors

A molecular explanation for high FDG uptake in cancerous tissues is the overexpression of GLUT1, the molecule reported to be responsible for FDG uptake in various cancers [20,26]. Glucose uptake ability as assessed by FDG-PET was significantly correlated with the doubling time of tumors [27] because increased uptake can provide additional energy to support tumor growth. Yamada et al. [7] determined from immunohistochemistry that GLUT1 expression was an important factor for FDG uptake and also a prognostic tool for gastric cancer. Alakus et al. [3] similar reported that FDG uptake in gastric cancer is dependent on the degree of GLUT1 staining. Our immunohistochemical staining also showed strong GLUT1 expression in cell membranes, as well as GLUT1 mRNA expression 3.3-fold greater in tumors than the surrounding mucosa; however, Spearman’s correlation analysis did not find a relationship between GLUT1 expression and SUV. HK2 also plays an important role in FDG catabolism, with its overexpression significantly associated with SUV in malignant tumors [15,28]. We also found HK2 overexpression in gastric cancer tumors, but there was again no correlation between HK2 expression and SUV. Other complicated mechanisms, such as blood flow, accumulation of inflammatory cells, and cellularity might be also contribute to the intensity of FDG uptake based on malignant energy demand [20].

### Hypotheses of the increased glucose uptake in tumor

Two major hypotheses have been presented to explain the increased glucose uptake in cancerous tissue, either that enhanced glucose consumption is associated with tumor proliferative activity [12,13] or that tissue hypoxia induces anaerobic glycolysis to increase glucose metabolism [14]. Our results indicate that FDG uptake associated significantly with hypoxia, reflected by HIF1α expression, but not with proliferative activity, reflected by PCNA expression; these gastric cancer findings correspond to our previous report on colorectal cancer [20]. Rapid cancer growth induces a hypoxic environment in tumors. HIF1α acts as a sensor for hypoxic stress and upregulates angiogenic factors and promotes transcription of several genes, including glucose transporters and glycolytic enzymes such as GLUT1 and HK, for tumor survival [29]. HIF1α may also be involved with oncogenic alterations to glucose metabolism because it activates cancer-related gene transcription and affects pathways such as angiogenesis, cell survival, glucose metabolism, and cell invasion [30]. HIF1α overexpression has been associated with increased patient mortality rates in several cancers, while inhibited expression reduced tumor growth in an in vitro study [30]. HIF1α could thus play a central role in cancer progression that FDG uptake represents.

### Histological differences in the expression of glucose metabolism-related proteins

The non-intestinal gastric cancers, signet ring cell carcinoma and mucinous carcinoma, presented a very low FDG uptake compared to their intestinal counterparts due to low GLUT1 expression [1,3,7,8]. Berger et al. reported that FDG-PET revealed an unusually high percentage (41%) of false-negative results in carcinoma with mucin. There was a positive correlation of FDG uptake with tumor cellularity but a negative correlation with the amount of mucin [31]. Therefore, non-intestinal gastric cancers, which have characters of low cellularity and/or high mucin content, do not show high FDG uptake. Alakus et al. has reported that over expression of GLUT1 in papillary/tubular adenocarcinoma and signet ring cell carcinoma was 94% and 24%, respectively [3]. Our results also indicate that GLUT1 expression in non-intestinal cancers was lower than in intestinal cancers. However, the reason why such aggressive cancers showed low GLUT1 expression is unknown. A previous study found that glutamine metabolism is upregulated in gastric cancer [32]. Gastric cancer cells use glutamine as an energy source in a hypoxic tumor microenvironment, which may eliminate the necessity for glucose transport. This metabolic alteration accompanied with malignant transformation has been reported in other cancers [33]. Interestingly, a glutamine-based PET is being developed; if successful, this contradiction could be disproved in the future.

On the other hand, HIF1α expression correlated with SUV in both types, although a more significant correlation was seen in non-intestinal specimens. The non-intestinal tumors may have been influenced more by hypoxia derived from tumor fibrosis due to a scattering tumor growth pattern than hypoxia due to increased tumor size. Further research will be needed to determine the exact reason.

### Limitations of this study

There are several limitations in our study. First, we examined 50 cases of gastric cancer patients. The fewness of cases affects the statistical analysis and makes it difficult to get firm results in association of FDG uptake and the expression of the proteins. Second, we could not exclude the possibility of contribution of physiological FDG uptake in normal stomach on cancerous lesion. Finally, our results did not show the direct physiological relationship between HIF1α as a marker of hypoxic condition and FDG accumulation.

## Conclusions

The usefulness of FDG-PET in the detection of malignant tumors or prediction of prognoses has been widely reported. However, our results indicate that the degree of FDG accumulation does not always suggest a prognosis in gastric cancer. This study is the first to show the correlation by evaluating FDG uptake in a quantitative manner. Upregulation of glucose transport due to increased GLUT1 expression was not an explanation for the different FDG uptakes observed, although tumor hypoxia and HIF1α expression may provide a reasonable mechanism. Further investigation is needed to confirm these results, but metabolic alternation through HIF1α induction in tumor hypoxia could increase FDG uptake in gastric cancer.

## Competing interests

The authors declare that they have no competing interests.

## Authors’ contributions

RT: Analyzing data, experimental work, and drafting article. KI: Conception, design, experimental work, and acquiring data. YY: Acquiring and analyzing data of FDG-PET. RK: Acquiring and analyzing data of FDG-PET. HM: Acquiring clinical data. TM: Revising the manuscript, and statistical analysis. YS: Enhancing its intellectual content. All authors read and approved the final manuscript.
